# Pre-contoured Plate Fit Assessment for Acetabular Fractures

**DOI:** 10.1007/s10439-025-03760-9

**Published:** 2025-05-26

**Authors:** Willemina A. van Veldhuizen, Nick Assink, Richte C. L. Schuurmann, Reza Firoozabadi, Kaj ten Duis, Jean-Paul P. M. de Vries, Jelmer M. Wolterink, Frank F. A. IJpma

**Affiliations:** 1 Department of Surgery, University Medical Centre Groningen, University of Groningen, Hanzeplein 1, 9700 RB Groningen, The Netherlands; 2 3D lab, University of Groningen, University Medical Centre Groningen, Groningen, the Netherlands; 3 Multimodality Medical Imaging Group, Technical Medical Centre, University of Twente, Enschede, The Netherlands; 4 Department of Orthopaedic Surgery, Harborview Medical Centre, Seattle, WA USA; 5 Department of Applied Mathematics, Technical Medical Centre, University of Twente, Enschede, The Netherlands

**Keywords:** Acetabular fracture, Suprapectineal quadrilateral surface plate, Implant fitting

## Abstract

**Purpose:**

Insufficient fitting of pre-contoured plates for acetabular fractures might lead to inadequate fracture reduction, but it is unclear in which patients pre-contoured plates fit adequately. The aims of this study were to assess plate fitting in sex- and height-specific anatomical variations of the hemipelvis, and categorizing the outcomes as moderate or good fit.

**Methods:**

3D models from computed tomography (CT) scans were obtained from a dataset of 200 patients with an intact left hemipelvis. This dataset was divided into eight subgroups, based on sex and body height, and the plate was virtually fitted on each shape. Plate fitting was assessed by computing the iliopectineal line, the quadrilateral slope, and the root mean square distance (RMSD) between each coordinate of the plate and its closest coordinate on the hemipelvis.

**Results:**

The mean age was 56 ± 16 years, and the mean height was 173 ± 10 cm. All female pelves had a moderate fit, mainly because the plate length either exceeded the iliopectineal line length, or the plate’s anterior aspect was directed too ventrally. Three out of four male pelves had a good fit. Only the small height subgroup (<175 cm) showed a moderate fit due to the plate length exceeding the iliopectineal line length and a relatively high median RMSD value (1.5 [0.8–2.0] mm) in mid-section of the plate.

**Conclusions:**

In acetabular fracture surgery, both visual and quantitative evaluation of suprapectineal plate fitting in sex- and body height-specific subgroups showed moderate fitting in female pelvic shapes, indicating a need for substantial intraoperative bending. This suggests the need for different sizes and contours of future suprapectineal plates in acetabular fracture surgery.

## Introduction

Acetabular fractures are considered challenging fractures, due to the complex three-dimensional (3D) geometry of the pelvis and the displacement of fracture fragments in different directions [13]. The standard treatment for displaced acetabular fractures is open reduction and internal fixation (ORIF), aiming for anatomical reduction to preserve long-term functionality of the hip joint and decrease the risk of post-traumatic arthritis. To minimize this risk, and ultimately reduce the need to converse to a total hip arthroplasty (THA), adequate fracture reduction and implant positioning are needed [8, 22].

Prior to surgery, it is difficult to determine if a pre-contoured osteosynthesis plate fits the patient’s specific pelvic anatomy, due to differences in fracture type and patient’s sex and body height. Insufficient fitting of a pre-contoured plate might lead to inadequate fracture reduction and lack of appropriate support of the displaced fracture fragments [4, 10, 20]. Multiple bending manoeuvres of a plate during surgery are suboptimal due to extended surgery time, possibly weakening of the plate material, and a higher risk of complications [14, 18, 23, 24]. Patient-specific plates provide a better fit with the patient’s anatomy but require more (technical) expertise, costs and production time compared to a conventional off-the-shelf pre-contoured plate [15]. Currently, it is not entirely clear for which sizes and shapes of the hemipelvis an off-the-shelf plate fits well.

To treat complex acetabular fractures, including anterior column, anterior column with posterior hemi-transverse, and associated both column and quadrilateral surface fractures, the pre-contoured suprapectineal quadrilateral surface plate (QLS plate) can be used [4, 25]. The main challenge of fractures involving the quadrilateral surface is achieving anatomical fracture reduction and keeping the fracture reduced using implants with a proper fit [4]. There is no one-size-fits-all plate that matches the variety of anatomies and fracture patterns of each patient with an acetabular fracture [9, 14]. Moreover, Cha et al. (2023) described an improper fitting of the QLS plate with pelvic shapes in the Korean population, experiencing insufficient restoration of the superior dome or the quadrilateral surface [4].Our clinical experience indicates that the plates commonly used in acetabular surgery for maintaining reduction and fixation of bone fragments often do not fit optimally given the wide variation among patients, particularly with respect to sex, body height, and physique. Besides that, previous literature showed size- and sex-related differences in acetabular geometry [2, 3, 19]. Hence, knowledge about the anatomical shape differences of the hemipelvis within a specific population is needed to evaluate the QLS plate-specific region of the hemipelvis.

The primary aim of this present study was to assess plate fitting in sex- and height-specific anatomical variations of the hemipelvis, using both visual assessment and quantitative analysis. The secondary aim was to clinically interpret these fittings, categorizing the outcomes as either moderate or good fit. This resulted in a clinical assessment tool that provides surgeons with recommendations on plate fitting based on patient characteristics known prior to surgery. Surgeons would then be able to decide to use a non-contoured plate strategy or be prepared to contour the plate prior to implantation.

## Methods

This retrospective, diagnostic imaging study was reviewed, and a waiver was provided by the Medical Ethics Review Committee of our medical centre, no: 202000092. This study is reported following Strengthening the Reporting of Observational Studies in Epidemiology (STROBE) guidelines and was performed in line with the Declaration of Helsinki [7].

### Study population

The study population comprised 200 patients treated at the department of surgery, 100 male and 100 female patients, with CT scans of intact hemipelves in adult patients. Inclusion criteria were the availability of a CT scan; age > 18 years; the presence of an intact and complete left hemipelvis; and the availability of demographic information including age, sex, height and weight. Exclusion criteria were as follows: (1) (a history of) pelvic fractures; (2) congenital hip defects; (3) and hip implants in situ. In the previous work, a first initial registration was performed to obtain a mean shape from all 200 3D shapes in the dataset to avoid bias from choosing a random template [21]. The 3D shapes of these hemipelves were registered, as previously described, to the mean shape by means of iterative closest point (ICP) algorithm to ensure anatomical point-to-point correspondence between all shapes [21]. This correspondence enables the calculation of a mean shape for a specific subset of patients.

### Construction of shapes

In this study, nine different shapes were constructed, namely the mean shape of the whole population (*n* = 200) and the mean shapes of eight subgroups of the population stratified by sex and height. Body height was used to subdivide each sex-specific group into four subgroups, as this information can be easily obtained during a consultation without the need for CT imaging or volumetric data. Correlation between a patient’s body height and hemipelvis volume was computed by calculating the Pearson correlation coefficient. Distribution of height and weight was comparable to distributions found in the Dutch population [26, 27]. First, the total dataset was divided by sex, resulting in two groups of 100 patients each. Accordingly, each sex-specific group was divided into four subgroups, based on the median and the quartiles of sex-specific body height, with Q1 representing shorter patients and Q4 taller patients. Some patients have a body height exactly corresponding to the cut-off median and quartile values. Patients with a body height matching the cut-off value were allocated to the subsequent group, resulting in a different number of patients in each quartile. For example, the cut-off value for the division of female Q1–Q2 was 163 cm. Female patients with this specific body height were allocated to the Q2-group. For each sex- and body height-specific subgroup, male and female Q1–Q4 groups, one mean shape was calculated, resulting in eight template shapes.

**Fig. 1:**
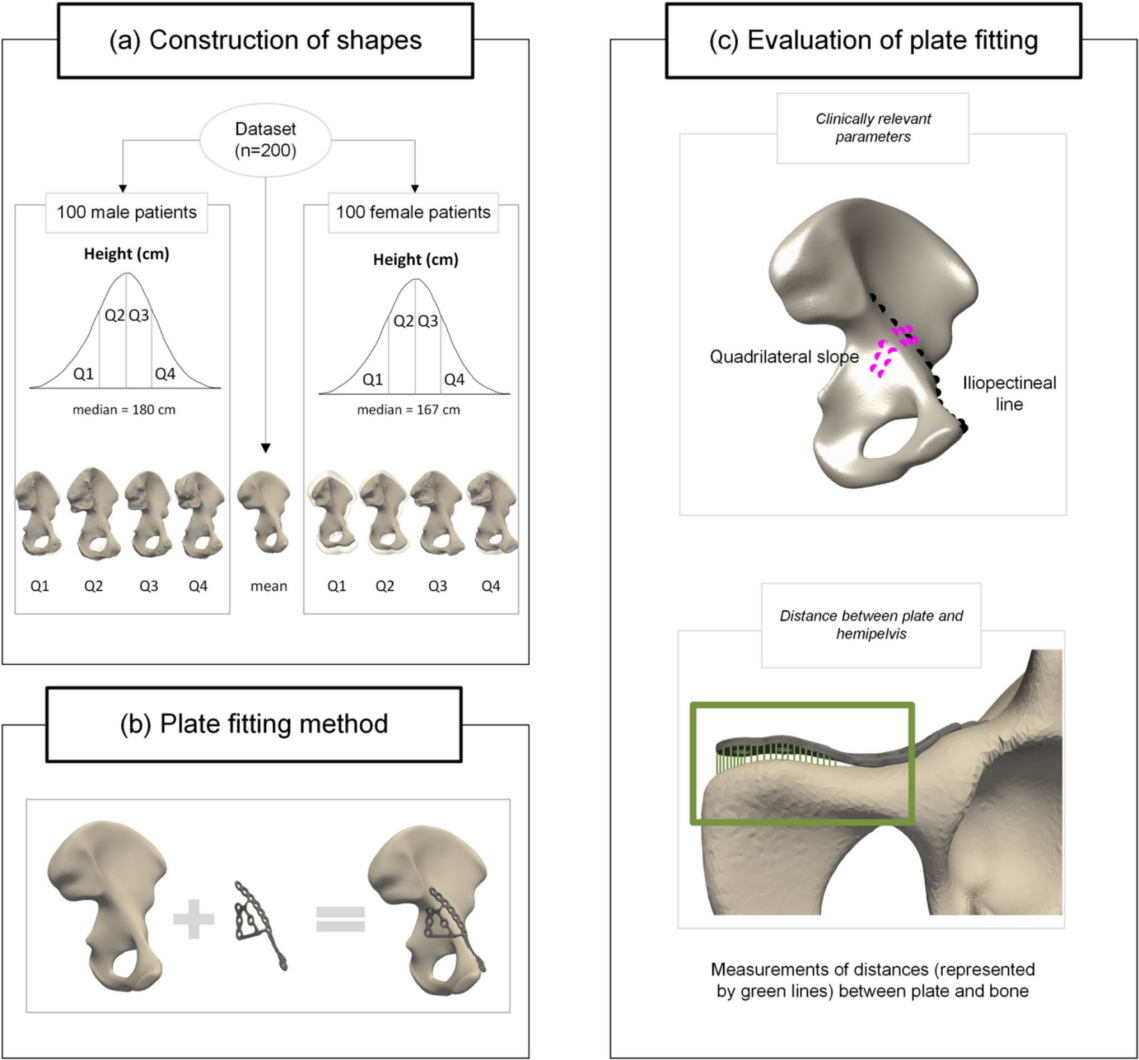
Workflow from construction of shapes to plate fitting analysis. **a** A dataset of 200 patients was used to construct different shapes. A mean shape was constructed from all 200 patients. Accordingly, the dataset was divided by sex, resulting in 100 female and 100 male patients. Then, for each sex-specific group, four subgroups were defined based on the patients’ body height distribution (quartile 1-4; Q1-Q4) within each sex-specific group. **b** Plate fitting of the quadrilateral suprapectineal (QLS) plate was performed on all nine hemipelvis shapes. **c** Evaluation of plate fitting by computation of clinically relevant parameters, including iliopectineal line (black dots) (1) length, (2) radius, and (3) the quadrilateral slope (pink dots), and distance between the plate and the hemipelvis, represented by the green lines between the plate and the bone

The workflow of the construction of shapes and plate fitting analysis is shown in Fig. 1.

### Plate fitting procedure

**Fig. 2:**
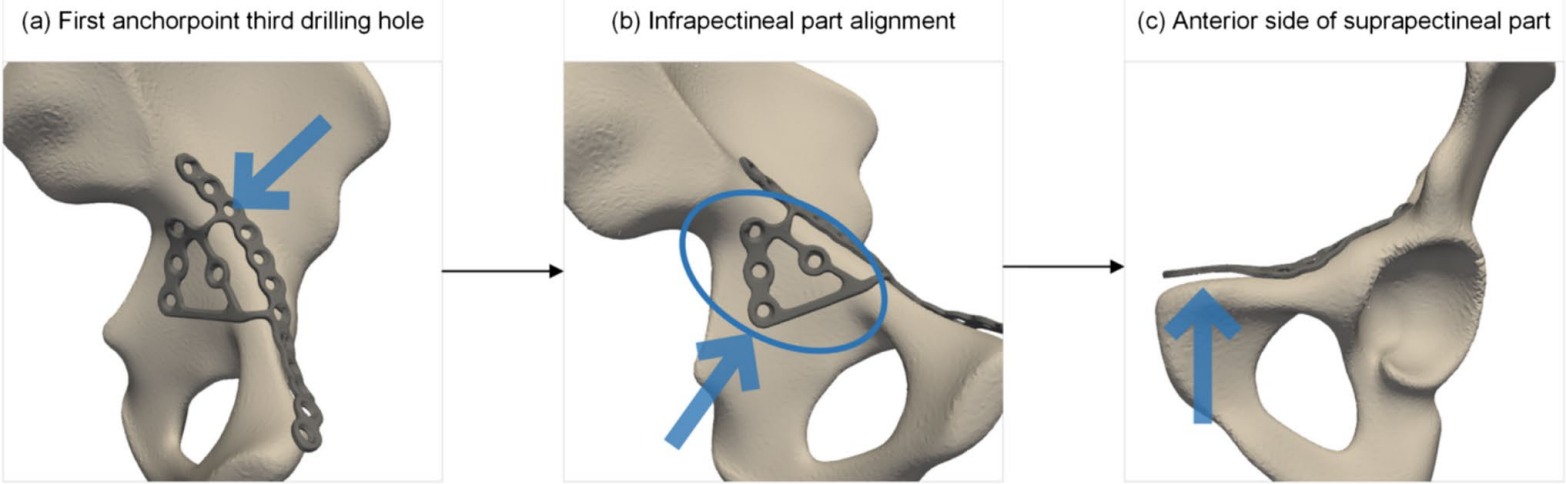
Plate fitting performed according to virtual surgical operative technique as described in the surgical guide by Stryker [25]. **a** The first anchor point of the plate is the third hole of the suprapectineal part of the plate. **b** The infrapectineal part of the plate was aligned utilizing this anchor point. **c** The anterior part of the plate can be finetuned and adjusted to align the anterior part of the hemipelvis.

On each of these nine shapes, the QLS plate was manually fitted in 3-matic software (Version 18.0, Materialise, Leuven, Belgium). Plate fitting was performed by consensus of an experienced technical physician and biomedical engineer (N.A.; W.V) and these fittings were evaluated by an experienced orthopaedic trauma surgeon with sub-specialty in pelvic and acetabular surgery (F.IJ.). The fitting procedure followed the principles of the provided instructions of the QLS plate [25]. The first anchor point of the plate with the hemipelvis is the third hole of the suprapectineal part of the plate (Fig. 2a). Accordingly, the infrapectineal plate was aligned with the hemipelvis to the greatest extent possible, utilizing the third hole as the anchor point (Fig. 2b). Then, the anterior part of the suprapectineal plate was finetuned and adjusted to align with the anterior part of the hemipelvis (Fig. 2c). Since virtual bending of the plate was not possible, plate fitting was conducted with the pre-contoured plate shape. To avoid possible intersection of the plate with the bone, we used the surface of the plate for plate fitting

### Evaluation metrics of plate fitting

**Fig. 3:**
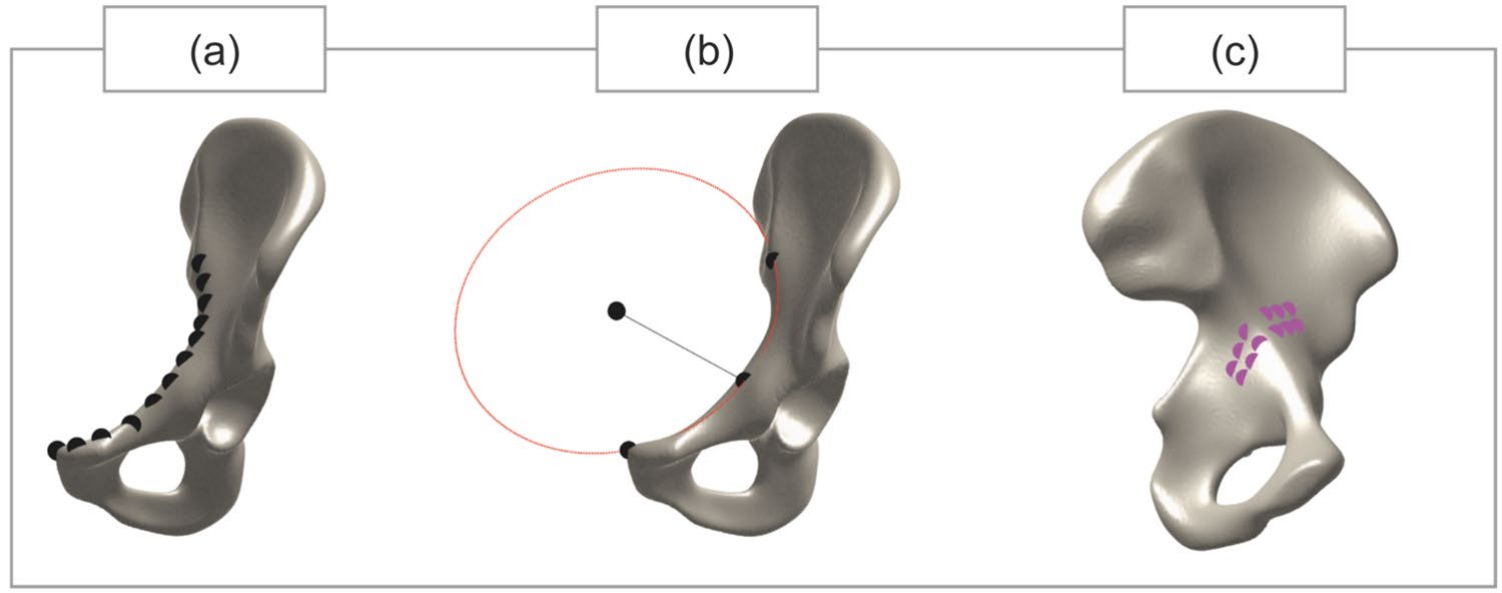
Visualization of the computation of the clinically relevant parameters. **a** Iliopectineal line length, **b** iliopectineal line radius and **c** quadrilateral slope

Clinically relevant parameters, including the iliopectineal line (1) length, (2) radius, and (3) the quadrilateral slope (Fig. 3), were defined on both the plate and the shape.

Computation of these parameters was performed in MATLAB (MATLAB 2023a, The MathWorks, Inc., Natick, MA, USA). First, coordinates were manually located on the mean shape, and the corresponding indices were saved. Accordingly, coordinates were automatically retrieved on other shapes of interest. The iliopectineal line length was computed by connecting the black coordinates as shown in Fig. 3a, and a length for each shape was computed. To compute the quadrilateral slope, 6 coordinates were selected twice (twelve pink dots in Fig. 3c), and two planes were created from each set of 6 coordinates. The angle between these two planes was calculated and reported as the quadrilateral slope. The iliopectineal radius, as shown in Fig. 3b, was computed by taking the first, middle and last coordinate of the black dots shown in Fig. 3a, and a circle was fitted through these three coordinates. The radius of the fitted circle was reported as the iliopectineal line radius

**Fig. 4:**
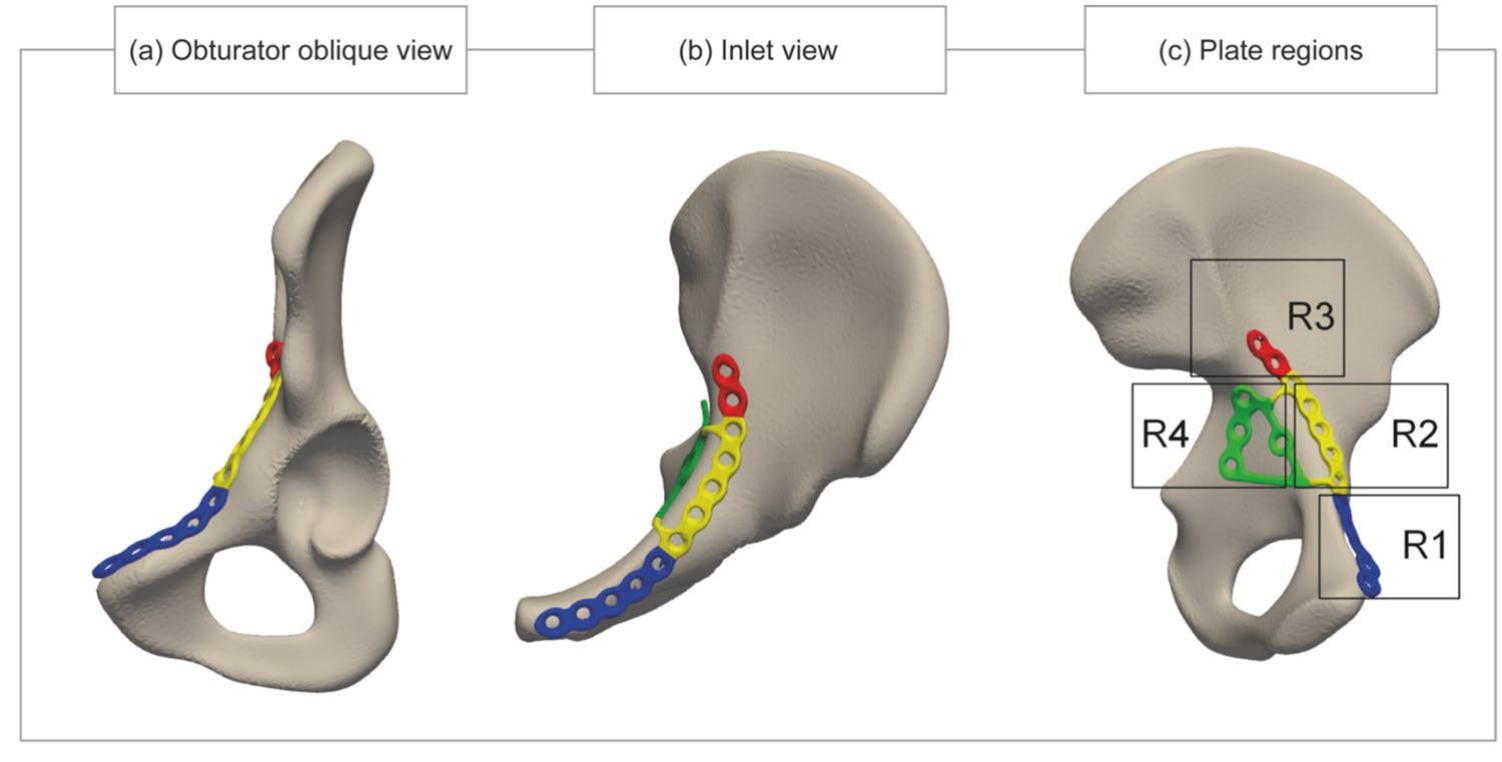
**a** Obturator oblique view and **b** inlet view of the mean shape and the fitted plate. **c** To compute the root mean square distance (RMSD) for specific parts of the plate, four regions of the plate were defined. Region 1 (blue) represents the anterior part of the plate, region 2 (yellow) represents the suprapectineal part of the plate, region 3 (red) represents the posterior part of the plate, and region 4 (green) represents the infrapectineal part of the plate

After plate fitting, the Euclidean distance was computed between each coordinate of the plate and its closest corresponding coordinate on the hemipelvis, aiming to quantify the accuracy of the fit. For all coordinates, this distance was obtained, and subsequently, the root mean square distance (RMSD) of the whole plate was calculated. Additionally, four distinct regions of the plate were defined to assess local plate fitting. Figure 4 shows these four regions as region 1 (R1) the anterior part of the plate (blue), region 2 (R2) the suprapectineal part of the plate (yellow), region 3 (R3) the posterior part of the plate (red), and region 4 (R4) the infrapectineal part of the plate (green) [25]. As regions 2 and 4 of the plate must align accurately with the bone to provide sufficient support, the RMSD values in these regions should be as low as possible. Accordingly, the RMSD values of these two regions are utilized for the evaluation of plate fitting.

Finally, a measure for the fit was defined in consensus with the authors as moderate or good, based on visual interpretation, the RMSD of regions 2 and 4, and the clinical parameters. A moderate fit requires significant additional pre- or intraoperative bending adjustments, whereas a good fit allows the pre-contoured plate to be utilized without bending adjustments.

Data were presented as mean ± standard deviation for demographic data and median (interquartile range IQR; 25th and 75th percentile) for RMSD values.

## Results

### Study population

The mean age was 56 ± 16 years, and the mean height and weight were 173 ± 10 cm and 81 ± 19 kg, respectively. Table 1 shows the age, height, weight and hemipelvis volume distribution for the shapes of the eight defined subgroups. The median height (range) of the male subgroup was 180 (163–196) cm, and the median height (range) of the female subgroup was 167 (146–194) cm. A strong correlation was found between body height and hemipelvis volume (R = 0.73).

**Table 1:** Demographic information of the eight subgroups based on sex- and height

	Height (cm)	Age (years)	Weight (kg)	Hemipelvis volume (× 10^4^ mm^3^)
	*Male subgroup*			
Q1 *(n = 23)*	171 (163–175)	62 ± 15	84 ± 17	37.6 ± 5.0
Q2 *(n = 21)*	177 (176–179)	57 ± 18	81 ± 13	41.1 ± 6.0
Q3 *(n = 27)*	181 (180–184)	50 ± 18	88 ± 19	42.5 ± 5.2
Q4 *(n = 29)*	188 (185–196)	49 ± 15	95 ± 20	45.1 ± 5.8
	*Female subgroup*			
Q1 *(n = 25)*	160 (146–162)	58 ± 15	67 ± 15	28.7 ± 4.6
Q2 *(n = 23)*	165 (163–166)	59 ± 16	69 ± 18	30.9 ± 3.7
Q3 *(n = 24)*	168 (167–169)	61 ± 11	74 ± 14	32.8 ± 3.4
Q4 *(n = 28)*	175 (170–194)	52 ± 13	83 ± 15	34.6 ± 3.4

*Values are presented as mean ± standard deviation, except for height, which is presented as median (range). The eight subgroups are defined by four different quartiles from the distribution of height in the sex-specific group. For example, the first quartile describes those patients whose heights belong to the first quartile, the second quartile the next number of patients whose heights fall between the 25^th^ quartile and the median value, etcetera. Q1 = quartile 1, etc.

**Fig. 5:**
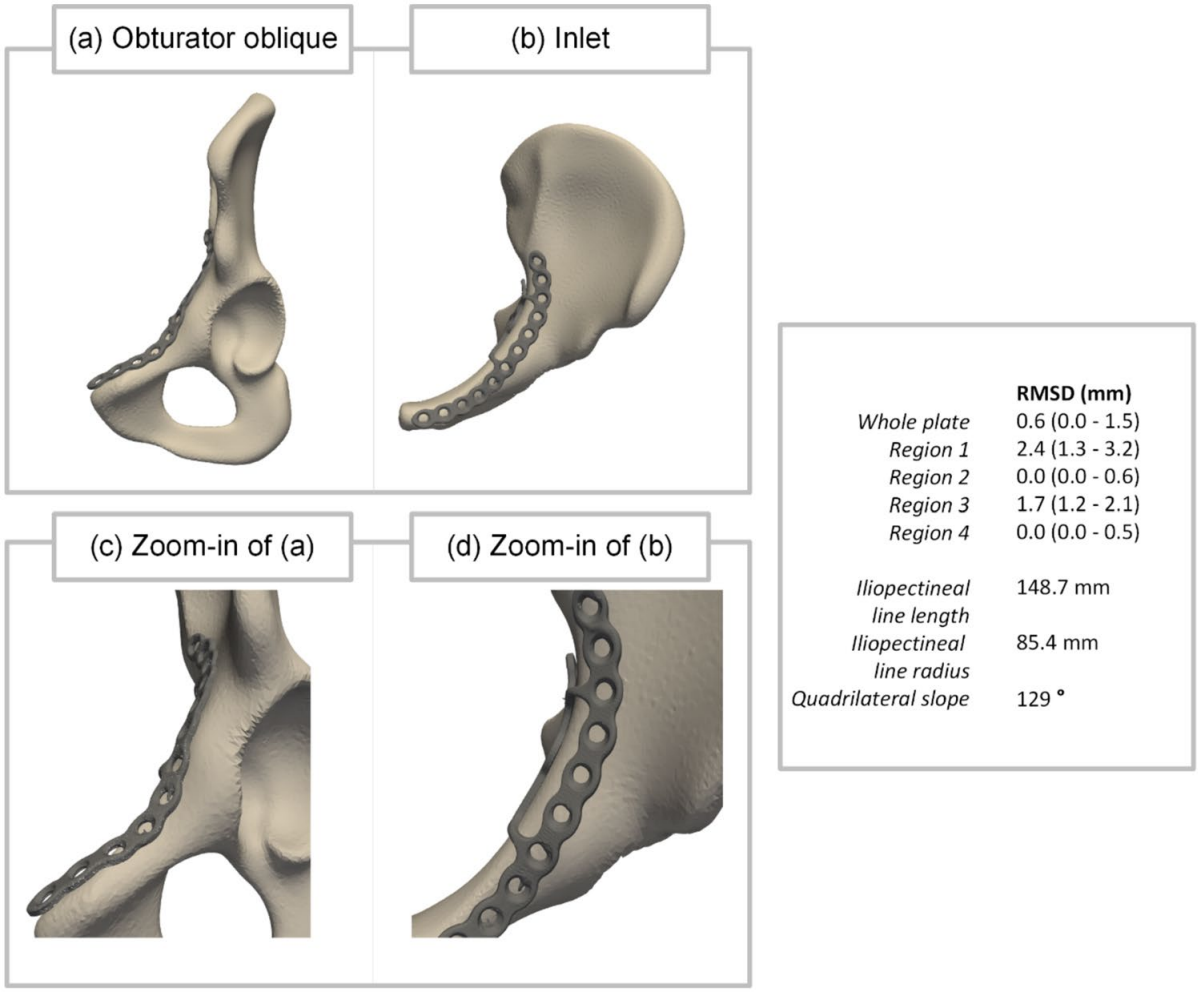
Visualization of plate fitting for the mean shape **a** obturator oblique view and **b** inlet view, **c** a zoom-in of the obturator oblique view **d** and a zoom-in of the inlet view. The root mean square distance (RMSD) of the whole plate with the mean shape is 0.6 (0.0–1.5) mm. In regions 2 and 4, an RMSD of 0.0 (0.0–0.6) mm and 0.0 (0.0–0.5) mm was reported, respectively

Figure 5 shows the plate fitting of the mean shape, encompassing the mean pelvic shape of all males and females together. In regions 2 and 4, RMSD values of 0.0 (0.0–0.6) mm and 0.0 (0.0–0.5) mm were reported, respectively.

Table 2 shows the values of the three clinically relevant parameters (visualized in Figure 3) for the QLS plate, the mean shape and the shapes of the eight subgroups. The values for these three parameters are comparable between the QLS plate and the mean shape. The male Q2 subgroup has a higher value for the iliopectineal line length compared to the QLS plate and mean shape (166.1 mm vs. 144.0 mm and 148.7 mm, respectively). The female Q1 and Q2 subgroups show lower values for the iliopectineal line lengths (130.4 and 137.8 mm, respectively) compared to both the QLS plate and the mean shape. Quadrilateral slope values are similar in the different subgroups and slightly deviate from the value of the QLS plate.

**Table 2:** Results of clinically relevant parameters for the implant and the mean shape and the shapes of the eight subgroups

	Iliopectineal line length (mm)	Iliopectineal line radius (mm)	Quadrilateral slope (°)
QLS plate	144.0	87.4	111.5
Mean shape	148.7	85.4	129.0
*Male group*			
Q1 *(163–175 cm)*	142.5	82.7	129.5
Q2 *(176–179 cm)*	166.1	91.5	138.0
Q3 *(180–184 cm)*	148.8	88.1	138.3
Q4 *(185–196 cm)*	150.5	79.7	127.1
*Female group*			
Q1 *(146–162 cm)*	130.4	71.5	125.5
Q2 *(163–166 cm)*	137.8	80.1	121.9
Q3 *(167–169 cm)*	156.3	89.5	127.1
Q4 *(170–194 cm)*	149.5	75.9	133.1

*The eight subgroups were defined by four different quartiles from the distribution of height in the sex-specific group. For example, the first quartile describes those patients whose height belongs to the first quartile, the second quartile the next patients whose height falls between the 25th quartile and the median value, etc. QLS = quadrilateral surface; Q1 = quartile 1, etcetera.

Table 3 shows the RMSD of the whole plate and the four subregions of the plate for all nine shapes. The median RMSD between the whole plate and the mean shape was 0.6 (0.0–1.5) mm.

**Table 3:** Results of root mean square distance (RMSD) between the plate and the hemipelvis of the mean shape and the shapes of the eight subgroups

	Whole plate (mm)	Region 1 (mm)	Region 2 (mm)	Region 3 (mm)	Region 4 (mm)
Mean shape	0.6 (0.0–1.5)	2.4 (1.3–3.2)	0.0 (0.0–0.6)	1.7 (1.2–2.1)	0.0 (0.0–0.5)
*Male group*					
Q1 *(163–175 cm)*	1.4 (0.7–2.3)	2.0 (1.3–3.2)	1.5 (0.8–2.0)	2.6 (1.5–3.3)	0.8 (0.4–1.5)
Q2 *(176–179 cm)*	1.5 (0.8–4.9)	7.2 (5.0–9.7)	0.8 (0.5–1.5)	4.7 (3.2–5.6)	0.9 (0.6–1.4)
Q3 *(180–184 cm)*	1.6 (0.7–4.4)	8.8 (5.8–13.2)	0.9 (0.0–1.5)	4.0 (3.0–4.6)	0.9 (0.6–1.4)
Q4 *(185–196 cm)*	0.9 (0.5–1.4)	2.2 (1.3–2.9)	0.9 (0.6–1.3)	0.9 (0.6–1.1)	0.6 (0.0–0.9)
*Female group*					
Q1 *(146–162 cm)*	1.3 (0.6–2.9)	3.8 (2.2–5.9)	1.6 (1.1–2.7)	0.0 (0.0–0.3)	0.8 (0.5–1.5)
Q2 *(163–166 cm)*	0.7 (0.0–1.6)	3.3 (1.6–5.5)	0.5 (0.0–0.7)	0.8 (0.3–1.3)	0.4 (0.0–1.1)
Q3 *(167–169 cm)*	1.9 (0.7–3.0)	5.1 (2.4–5.9)	0.2 (0.0–0.7)	2.5 (1.7–3.3)	2.0 (1.2–2.5)
Q4 *(170–194 cm)*	0.8 (0.3–1.8)	2.0 (0.7–3.6)	0.7 (0.0–1.1)	2.9 (1.8–3.6)	0.6 (0.0–0.9)

*Values are represented as median (IQR; 25th and 75th percentile). The eight subgroups were defined by four different quartiles from the distribution of height within the sex-specific group. For example, quartile 1 (Q1) includes patients whose height is below the 25th percentile, quartile 2 (Q2) includes patients whose height is between the 25th percentile and the median, etc. Region 1 (R1) corresponds to the blue part of the plate, region 2 (R2) to the yellow part of the plate, region 3 (R3) to the red part of the plate and region 4 (R4) to the green part of the plate (Fig. 4c shows the colours assigned to different regions of the plate)

**Fig. 6:**
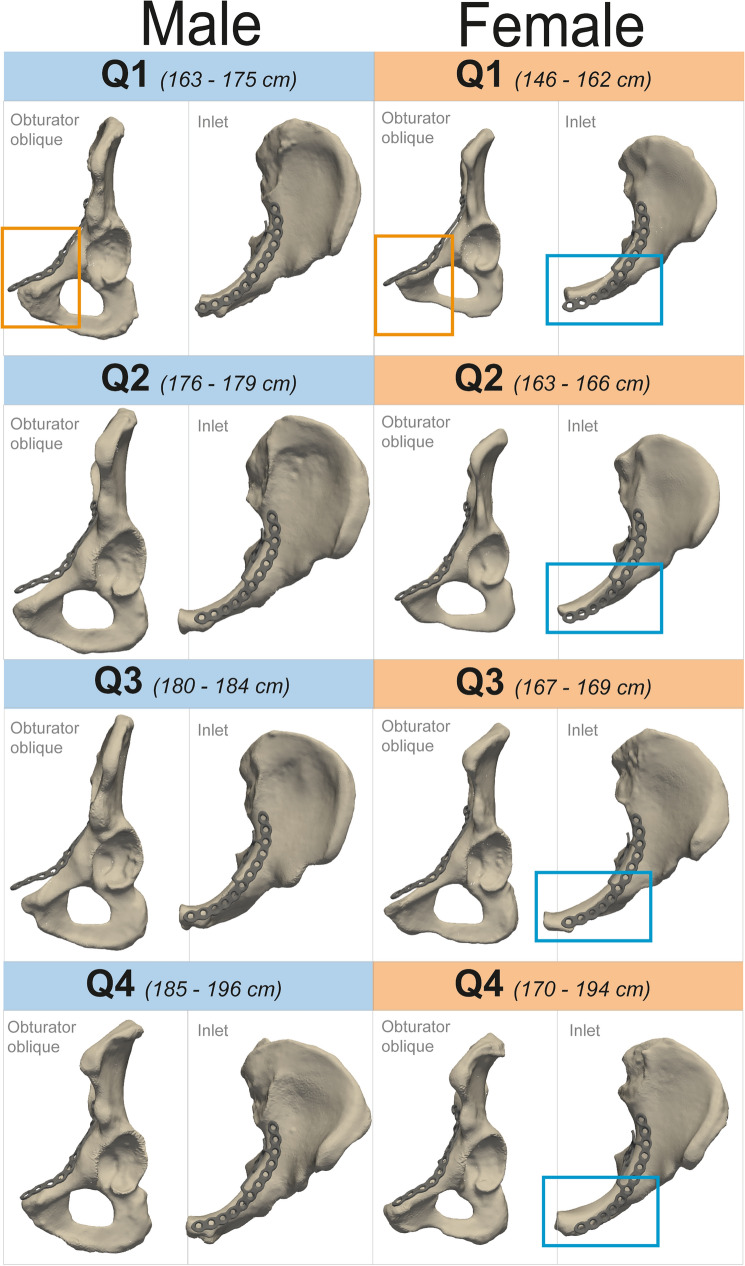
Assessment tool for clinical interpretation of plate fitting of the male and female subgroups. When a patient is presented with an acetabular fracture, he or she could be allocated to a sex-specific group. Accordingly, the patient’s height determines in which Q-subgroup the patient belongs. For each subgroup, the plate fitting is evaluated by means of the visual inspection, with bounding boxes indicating reasons for a moderate fit. A moderate fit requires significant additional pre- or intraoperative bending adjustments, whereas a good fit allows the pre-contoured plate to be utilized without bending adjustments. An orange colour box highlights the plate length exceeding the length of the iliopectineal line and a blue coloured box highlights ventral protrusion of the plate relative to the iliopectineal line of the hemipelvis, both contributing to a moderate fit assessment

Based on the results obtained in this study, we propose an assessment tool (Fig. 6), from which the surgeon can determine whether an off-the-shelf plate will be sufficient without bending adjustments (i.e. good fit) or if an off-the-shelf plate probably needs significant additional pre- or intraoperative contouring (i.e. moderate fit), given a patient’s sex and body height.

For the male subgroups, the plate has a good fit with the Q2–Q4 shapes; the Q1 shape has a moderate fit, as the plate length extends beyond the pubic symphysis (144.0 mm versus 142.5 mm) and the median RMSD in region 2 is relatively high compared to the mean’s shape region 2 (1.5 mm versus 0.0 mm).

For the female subgroups, the Q1 shape shows the least favourable fit. The plate length exceeds the iliopectineal line length of this subgroup to a great extent (144.0 mm versus 130.4 mm). Moreover, the median RMSD of region 2 is higher than the median RMSD of region 2 of the mean shape (1.6 versus 0.0 mm). Regarding the Q2 shape, the fit is evaluated as moderate because the plate exceeds the iliopectineal line length of this shape (144.0 mm versus 137.8 mm). The main issue in the female Q3 and Q4 shapes is that the anterior part of the plate is directed too ventrally (Figure 6 2nd column).

## Discussion

The primary aim of this study was to assess suprapectineal acetabular plate fitting in sex- and height-specific anatomical variations of the hemipelvis, using both visual assessment and quantitative analysis. The accuracy of the fit on the mean shape was very good; low RMSD values were observed for the plate overall, and specifically for regions 2 (suprapectineal part of the plate) and 4 (infrapectineal part of the plate). Given that the plate aligns closely with the mean shape, it is probable that its design was based on such a mean shape. Visual and quantitative assessments of suprapectineal plate fitting revealed moderate fitting in females and shorter males (<175 cm), requiring significant intraoperative bending, while fitting was good in taller males.

The secondary aim was to clinically interpret suprapectineal plate fittings based on sex and body height, categorizing the results as either moderate or good fit. All four female height-based shapes (Fig. 6 (second column), Q1–4) had a moderate fit, mainly because the length of the plate either exceeded the iliopectineal line length of the shape or the anterior part of the plate was directed too ventrally (i.e. anterior aspect of the plate sits anterior to the anterior aspect of the superior rami). This can result in reduced plate functionality and inability to lateralize the quadrilateral surface. The moderate plate fitting observed across all female subgroups suggests the need for the development of a pre-contoured plate designed specifically for women. A good fit was observed in three out of four shapes (Fig. 6 (first column), Q2-4) of the male height-based shapes; only the Q1 shape (< 175 cm) showed a moderate fit due to the plate length exceeding the iliopectineal line length and a relatively high median RMSD value (1.5 [0.8–2.0] mm) in region 2 (Table 3). From these results, it can be concluded that the plate should be pre- or intraoperatively adjusted in patients whose height belongs to the Q1 subgroups (< 162 cm for women and < 175 cm for men). To serve the entire population, an alternative strategy could involve the development of plates in both a small and large size, considering differences in curvature of the plate.

A next step is to compare our findings with other studies, although similar research is limited. One of the few studies in this field is described by Osterhoff et al. (2019) [16]. They describe a method to quantify plate fitting to investigate whether there is a difference between an individual’s left and right hemipelvis [16]. They utilized the Stryker Orthopaedic Modelling and Analytics (SOMA) database (*n* = 516) and computed the distance between the plate and the bone for 2310 points, thereby covering every mm^2^ of the plate. They aimed to investigate the individual symmetry in the periacetabular surface, rather than to evaluate the plate fitting of a pre-contoured suprapectineal plate. Our study adds that it combines the distance measurements with other clinically relevant parameters, such as iliopectineal line length, and we provide recommendations for fitting based on a combination of visual assessment, the distance measurements, and the values for the clinically relevant parameters. Even though computations as described in this study are not common, previous work by Ahmad et al. (2007) showed an inferior performance of mechanical properties of a locking compression plate (LCP) if the distance between bone and plate exceeds 5 mm, whereas ≤ 2 mm is recommended [1]. Future work includes investigating which RMSD values are associated with suboptimal fracture fixation and clinical functional outcomes.

Based on our findings, we now have a clearer understanding of which subpopulations experience optimal or suboptimal fit with the suprapectineal acetabular plate. This insight allows surgeons to anticipate potential challenges in clinical practice. Specifically, they should be aware that pre-contouring of the plate may be necessary for female patients and shorter male patients. The adage ‘Plan your operation and operate your plan’ remains particularly relevant in orthopaedic trauma surgery, emphasizing the importance of 3D preoperative planning in these cases. Prior or during surgery, a reconstruction plate can be pre-contoured to fit the patient-specific anatomy, which can be performed by bending it on a template created from a 3D-printed hand-held fracture model [11]. Pre-contouring a plate before surgery may result in decreased operation time, less intraoperative bleeding, improved quality of fracture reduction and improved clinical outcomes [12, 17]. The primary advantage of the proposed assessment tool for clinical practice is its reliance on readily accessible patient information, specifically sex and body height. By utilizing these two straightforward parameters, an estimation of plate fitting can easily be derived. Consequently, the assessment tool can be broadly used by trauma surgeons without requiring sophisticated or advanced 3D software packages. The proposed assessment tool can be used to determine whether a patient could be treated with a perfectly fitting pre-contoured plate or whether significant manual contouring of a conventional plate would be necessary. In certain cases, particularly for women with a height of less than 162 cm, consideration of designing a patient-specific plate may be warranted.

This study has strengths and limitations. The first limitation of our study is related to the manual procedure of plate fitting. The plate was virtually fitted using 3-matic software (Version 18.0, Materialise, Leuven, Belgium), but this software does not provide tactile feedback during fitting. Therefore, in theory, it would be possible for the plate to intersect with the hemipelvis shape. To avoid a collision or intersection between the plate and the hemipelvis shape, we used the surface of the plate for plate fitting. Using a surface structure instead of the whole volume of the plate results in immediate visual feedback of the intersection of the plate and the bone. Moreover, during the virtual plate fitting procedure, the plate cannot be bent, but only translated and rotated. The second limitation is that only one specific, commonly pre-contoured plate was evaluated. The aim of this study was to provide a proof of concept for plate fitting assessment rather than to assess the fit quality of this specific plate. For future research, we suggest to evaluate the fitting accuracy of other acetabular plates. Furthermore, it should be noted that the proposed automatic methodology can be translated to evaluate other type of implants too, even outside the scope of acetabular surgery. The third limitation is that a European Caucasian population was included in this study, considering sex and body height differences within this population. Ethnicity also plays a role in shape differences, as described by Dudda et al. (2012) and Edwards et al. (2020) [5, 6]. Therefore, our results are mainly applicable to patients with a European Caucasian background. Nevertheless, for a clinically feasible workflow, it is recommended to assess plate fitting using a patient’s sex and height. 

In acetabular fracture surgery, both visual and quantitative evaluation of suprapectineal plate fitting in sex- and body height-specific subgroups showed moderate fitting in female pelvic shapes, indicating a need for substantial intraoperative bending. Plate fitting was moderate in shorter males (<175 cm) and good in taller males. This suggests the need for different sizes and contours of future models of suprapectineal plates in acetabular fracture surgery.
